# A scoping review of reporting ‘Ethical Research Practices’ in research conducted among refugees and war-affected populations in the Arab world

**DOI:** 10.1186/s12910-018-0277-2

**Published:** 2018-05-15

**Authors:** Jihad Makhoul, Rana F. Chehab, Zahraa Shaito, Abla M. Sibai

**Affiliations:** 1 Department of Health Promotions and Community Health, Faculty of Health Sciences, American University of Beirut, Beirut, Lebanon; 2 Purdue University, West Lafayette, Indiana USA; 3 Regional External Programs, American University of Beirut, Beirut, Lebanon; 4 Department of Epidemiology and Population Health, Faculty of Health Sciences, American University of Beirut, Beirut, Lebanon

**Keywords:** Ethics, Research, IRB, Refugees, War-affected populations, Review, Arab world

## Abstract

**Background:**

Ethical research conduct is a cornerstone of research practice particularly when research participants include vulnerable populations*.* This study mapped the extent of reporting ethical research practices in studies conducted among refugees and war-affected populations in the Arab World, and assessed variations by time, country of study, and study characteristics*.*

**Methods:**

An electronic search of eight databases resulted in 5668 unique records published between 2000 and 2013. Scoping review yielded 164 eligible articles for analyses.

**Results:**

Ethical research practices, including obtaining institutional approval, access to the community/research site, and informed consent/assent from the research participants, were reported in 48.2, 54.9, and 53.7% of the publications, respectively. Institutional approval was significantly more likely to be reported when the research was biomedical in nature compared to public health and social (91.7% vs. 54.4 and 32.4%), when the study employed quantitative compared to qualitative or mixed methodologies (61.7% vs. 26.8 and 42.9%), and when the journal required a statement on ethical declarations (57.4% vs. 27.1%). Institutional approval was least likely to be reported in papers that were sole-authored (9.5%), when these did not mention a funding source (29.6%), or when published in national journals (0%). Similar results were obtained for access to the community site and for seeking informed consent/assent from study participants.

**Conclusions:**

The responsibility of inadequacies in adherence to ethical research conduct in crisis settings is born by a multitude of stakeholders including funding agencies, institutional research boards, researchers and international relief organizations involved in research, as well as journal editors, all of whom need to play a more proactive role for enhancing the practice of ethical research conduct in conflict settings.

**Electronic supplementary material:**

The online version of this article (10.1186/s12910-018-0277-2) contains supplementary material, which is available to authorized users.

## Background

In the midst of the recent sociopolitical upheavals in the Arab region, in particular the Syrian crisis, research on refugees and war-afflicted populations has become of greater interest to the broader scientific community and humanitarian agencies. While research in situations of conflict and war is essential to guide programs and services, it may not be scientifically rigorous [1], and therefore, is rarely followed with publication plans. Reasons for the inadequate methodological rigor and for not giving publication plans in refereed journals serious attention include the overwhelming need to act swiftly and produce data that would support relief agencies in their humanitarian efforts and help save lives in emergencies. Additionally, the politicized nature of the issues addressed and the sensitivity of the findings to some parties, such as in the case of human rights violations, may preclude publicizing the findings or sharing them with larger audiences [2, 3]. But when research is carried out in such humanitarian contexts, responsible ethical conduct may be overlooked [4–6], thus posing adverse consequences on the research participants who may be vulnerable, marginalized or directly affected by armed conflicts. Considerations pertaining to the risk and benefits of the research, its neutrality and confidentiality, particularly in resource poor settings [7], improve participants’ diversity and representation, and enhance the quality of the research, its validity and utility [8].

Whilst guidelines for human subjects’ research exist in a number of academic disciplines, such as the social and medical fields, there is yet no single best ethical guideline for conducting research with refugees or war-affected populations in particular. A number of ways forward derived from practical research experiences in different humanitarian settings have been suggested to guide fieldwork and research in these contexts [2, 4, 5, 9]. Notable strides have been made on the part of international NGOs and UN agencies to guide humanitarian interventions. The Red Cross movement played an important role in developing codes of conduct for humanitarian aid by declaring four fundamental principles: humanity, independence, neutrality and impartiality [10]. More recently, Hunt and colleagues developed a framework for health professionals working with and alongside local and international actors and providing humanitarian assistance to individuals and communities [11].

The Arab world has long been beset by continuing armed conflict and political unrest which have caused considerable migration and population movements. More recently, several countries in the region have witnessed an escalation in the number of war-affected populations, both refugees and internally displaced populations (IDPs), as a result of waves of popular uprisings and armed internal conflicts [12]. Here, research practice is in its infancy [13] and is often not regulated by national or institution ethics review boards [14]. Only a few of the 22 Arab countries have highly functioning Institutional Review Boards (IRBs) entrusted with regulating human research conduct. This means that there are deficiencies in the knowledge base of researchers and regulators regarding research ethics, as well as inadequacies in the application of the principles of research ethics [15]. War-affected populations live in extremely vulnerable conditions enduring difficult social, economic and political hardships, and the need for ethical research guidelines that protect participants, and the researcher-researched relationship in these settings becomes even more imperative.

We examine in this study the extent of reporting ethical research conduct in articles on refugee and war-affected populations in the Arab world published between 2000 and 2013. The 2000 to 2013 study period was chosen because the majority of armed conflicts in the region occurred or started in this period, including the second Palestinian Intifada (2000), the Iraqi invasion (2003), and the waves of the Arab upheavals starting in Tunisia in 2011 and extending to the most devastating Syrian crisis [16, 17]. Earlier studies reviewing ethical research conduct in the literature have mainly addressed reporting practices in the biomedical field and clinical research and were confined to certain medical journals [18–20], or were focused on assessment criteria used for evaluating research ethics review [21]. However, no previous empirical research has examined the extent to which ethical research conduct is reported in studies among refugees and war-affected populations.

More specifically, this study aimed at mapping the extent of reporting ethical research conduct and factors that may promote or alternatively impede responsible conduct in published studies conducted with refugees and war-affected populations in the Arab World. The guiding research questions of our study are: 1) what is the extent to which three aspects of ethical research conduct (namely, research oversight, access to the community/research site, and informed consent and/or assent) are reported in research on IDPs, refugee or other war-affected populations in the Arab world; 2) do these vary by time and country of study; and finally, 3) how do reports of ethical conduct vary by study descriptors including participants’ characteristics, study discipline, methodology, profiles of author collaboration, funding source, journal type, and journal requirement for statement on ‘code of ethics’? Findings from this study contribute to the emerging field of ‘research on research ethics’ and are essential to relief agencies and researchers in their pursuit of ethically sound research in times of crisis.

## Methods

### Search strategy

This study followed Arksey and O’malley’s methods and framework of scoping reviews [22]. Scoping studies are emerging evidence-mapping tools that allow the review of a large scope of research output of various methods and quality to assess research gaps and opportunities and highlight areas for further in-depth analysis. The search strategy was put together by experts in the field of research ethics with an experienced librarian at the American University of Beirut, and was led by the keywords of interest to the research team. An electronic search of eight databases, namely PubMed, Web of Science, Google Scholar, Academic Search Complete, PROQUEST, EBSCO, JSTOR and MECAS was conducted for related articles published between 2000 and 2013. MECAS is an index of research, policy and scholarly discourse specific for countries and peoples of the Middle East, Central Asia and North Africa. Search terms relating to ethics were combined with terms related to refugees and war-affected populations (Additional file 1 shows this in more detail).

### Selection criteria

**Fig. 1:**
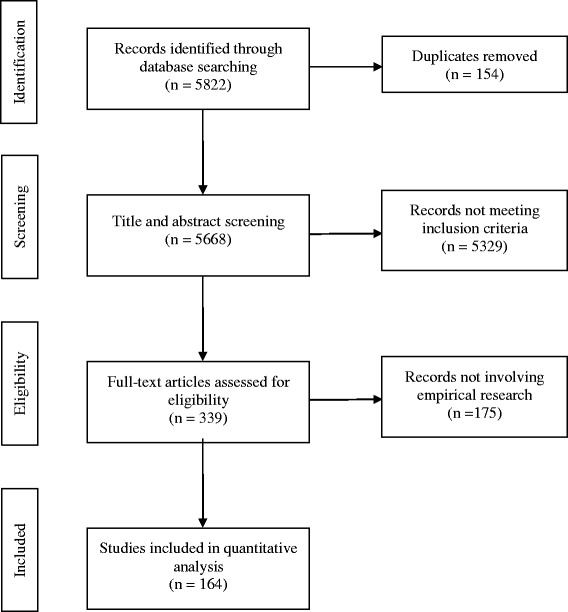
Flowchart of the screening process

The electronic search resulted initially in 5822 records for review. These publications were exported to Endnote, and after duplicate removal, 5668 records were retained for screening by title and abstract. A total of 5329 records were excluded as they did not meet the inclusion criteria. Published reports were included if they involved research with human subjects who were IDPs or refugees, if the study population pertained to one of the 22 Arab countries of the League of the Arab States, if they were peer reviewed articles, and if they were written in English. The remaining 339 publications were screened for full text and 175 publications were further excluded as these did not involve empirical research, thus yielding a total of 164 eligible articles included in our analysis (Fig. 1). Work was done in duplicates and in case the researchers did not agree on including or excluding a publication, the disagreement was resolved through discussion with the principal investigator.

### Charting the data

The research team developed a coding sheet based on study objectives and guided by their experience in research ethics. The coding sheet included details about the title of the article, authors’ names, the country and year of publication, and the research characteristics, including participants’ composition (internally displaced, refugees, others) and age groups (children, adults/older adults, all age groups). Details were also retrieved on the research characteristics, namely the discipline (public health, social, biomedical, others), methodology (quantitative, qualitative, mixed methods), as well as on other descriptors pertaining to first author affiliation (Arab, non-Arab), authors’ collaboration (national, regional, international, solo authorship), funding sources (national/regional, international, UN organizations, mixed, no funding), journal type (national, regional and international), and whether the journal online submission guidelines include a section on ‘code of ethics’ requiring authors to declare IRB approval and the manner of informed consent/assent (yes/no). Ethical research conduct, the main outcome of interest, was represented by three criteria, namely institutional approval and the body reviewing the research, (IRB/Research Ethics Committee [REC] or others such as the Ministry of Health/Education), access to the community/research site, and participants’ informed consent and/or assent. Details were recorded in a database and transferred later into a statistical package for analyses. Random checks were conducted for every 20th entry. All analysis was conducted using SPSS, and a *P*-value of < 0.05 was considered significant.

## Results

A steady increase in the number of published articles was noted over the study period (from 9 in 2000–01 to 60 in 2012–13) (Table 1). The majority of these publications came from Palestine (34.1%), followed by Lebanon, Sudan and Jordan (between 15.9 and 17.7%). The research participants in these articles included IDPs (55.5%) and refugees (39.6%) and involved mostly adults or older adults (65.9%). Most studies focused on issues related to public health (63%), and close to 57% used quantitative research methods. Nearly half of the articles were published with international co-authors (50.6%), 91% appeared in international journals, and the majority were published in journals (70.6%) that require a statement on ‘code of ethics’. Close to 50% of the papers did not mention any funding source. Among those that did, funding was mostly reported to be received from international sources (62%).

**Table 1:** Study descriptors

Variable		Categories		Frequency	Percent
Publication year		2000–01		9	5.5
		2002–03		16	9.8
		2004–05		14	8.5
		2006–07		20	12.2
		2008–09		21	12.8
		2010–11		24	14.6
		2012–13		60	36.6
Country		Palestine		56	34.1
		Lebanon		29	17.7
		Sudan		28	17.1
		Jordan		26	15.9
		Others^a^		25	15.2
Research participants	Composition	Internally displaced		91	55.5
		Refugees		65	39.6
		Others^b^		8	4.9
	Age groups	Adults/older adults		108	65.9
		Children		30	18.3
		All age groups		26	15.9
Research characteristics	Discipline	Public Health		103	62.8
		Social		34	20.7
		Biomedical		12	7.3
		Others^c^		15	9.1
	Methodology	Quantitative		94	57.3
		Qualitative		56	34.1
		Mixed methods		14	8.5
Other descriptors	First author affiliation	Arab		82	50.0
		Non-Arab		82	50.0
	Author collaboration	National		34	20.7
		Regional		5	3.0
		International		83	50.6
		Solo author		42	25.6
	Funding sources	International		51	31.1
		UN Organizations		5	3.0
		Mixed^d^		12	7.3
		No funding		7	4.3
		Not mentioned		81	49.4
	Journal type	National		2	1.2
		Regional		13	7.9
		International		149	90.9
	Journal submission guidelines	Code of ethics requirement		115	70.6
		No requirement		48	29.4
Ethical research conduct	Institutional approval	Yes	IRB/ REC	50	30.5
			Others^e^	29	17.7
			Total	79	48.2
		No/ Not mentioned		85	51.8
	Access to the community/research site	Yes		90	54.9
		Not mentioned		74	45.1
	Informed consent and/or assent	Yes		88	53.7
		Not mentioned		76	46.3

^a^Other countries include Algeria, Bahrain, Comoros, Djibouti, Egypt, Iraq, KSA, Kuwait, Libya, Mauritania, Morocco, Oman, Qatar, Somalia, Syria, Tunisia, UAE, Yemen and a combination of 2 or more Arab States together

^b^Other research populations include special interest groups such as health care professionals

^c^Example of other study types include anthropological studies

^d^Mixed funding includes concurrent funding from several sources such as the WHO and academic institutions

^f^Other institutional approval types include Ministry of Education and Ministry of Health

Institutional ethics approval to conduct the study was reported in 48.2% of the articles, with IRB/REC being the granting body in the majority of cases (Table 1). Close to 55% of the articles mentioned securing access to the research community/site, and 53.7% noted obtaining informed consent and/or assent from the research participants. Articles were significantly more likely to indicate access to the community site and to obtain consent/or assent from the study population when they reported obtaining institutional approval than those which did not (78.5% vs. 32.9 and 83.5% vs. 25.9%, respectively) (Table 2).

**Table 2:** Extent of reporting obtaining ‘access to the community/research site’ and ‘informed consent and/or assent’ stratified by reporting ‘institutional approval’

Reporting of	Institutional approval				
	Yes *N* = 79		No *N* = 85		*P*-value
	n	%	n	%	
Access to the community/research site (% yes)	62	78.5	28	32.9	< 0.001
Informed consent and/or assent (% yes)	66	83.5	22	25.9	< 0.001

**Fig. 2:**
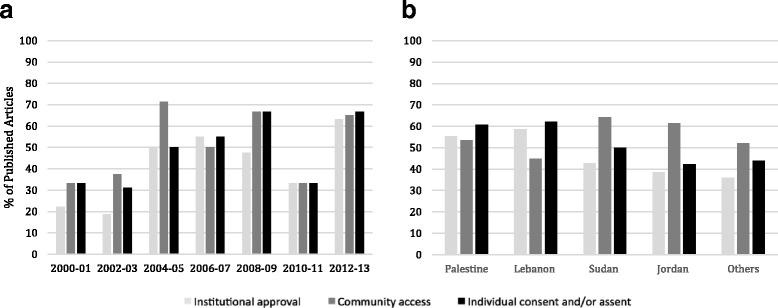
Reporting of ethical research conduct by time (**a**) and place (**b**)

The extent of adherence to the three outcome variables (institutional approval, access to the community/research site and informed consent and/or assent) was examined by time and place/country (Fig. 2). Except for a drop in 2010–11, an overall increase in the proportion of papers reporting ethical research conduct with time was noted. There was no clear differential in the distribution of reported ethical research conduct by country.

Table 3 shows variations in the reporting of ethical research conduct by study descriptors. The extent to which institutional approval was reported varied significantly with the composition of the study participants, being mostly reported in research that was conducted among IDPs (57.1%) compared to other types of study participants. It was also significantly more likely to be reported when the research was biomedical in nature (91.7%) as compared to public health or social science fields (54.4 and 32.4%, respectively), when the research employed quantitative methodologies (61.7%) compared to mixed methods or qualitative approaches (42.9 and 26.8%, respectively), and when the paper appeared in a journal requiring declaration of ‘code of ethics’ in its submission guidelines (57.4% vs. 27.1%). Articles that were least likely to report obtaining institutional approval were those that were sole-authored (9.5%), those that did not mention a funding source (29.6%) and those that were published in national journals (0%).

**Table 3:** Reporting of ethical research conduct by study descriptors

Independent variables		Categories	Institutional approval		Access to community/research sites		Informed consent and/or assent	
			% yes	*P*-value	% yes	*P*-value	% yes	*P*-value
Research participants	Composition	Internally displaced	57.1	0.031	59.3	0.150	60.4	0.136
		Refugees	40.0		52.3		46.2	
		Others	12.5		25.0		37.5	
	Age groups	Adults/ older adults	46.3	0.197	56.5	0.622	51.9	0.810
		Children	53.3		56.7		56.7	
		All age groups	50.0		46.2		57.7	
Research characteristics	Discipline	Bio-medical	91.7	< 0.001	91.7	0.001	83.3	< 0.001
		Public health	54.4		60.2		63.1	
		Social	32.4		41.2		35.3	
		Others	6.7		20.0		6.7	
	Methodology	Quantitative	61.7	0.001	67.0	< 0.001	64.9	0.001
		Qualitative	26.8		30.4		33.9	
		Both methodologies	42.9		71.4		57.1	
Other descriptors	First author affiliation	Arab	23.17	0.64	26.2	0.53	26.8	1.0
		Non-Arab	25.0		28.7		26.8	
	Author collaboration	National	55.9	< 0.001	55.9	< 0.001	70.6	< 0.001
		Regional	60.0		60.0		40.0	
		International	63.9		68.7		62.7	
		Solo author	9.5		26.2		23.8	
	Funding source	National/Regional	50.0	< 0.001	62.5	0.161	37.5	0.019
		International	64.7		58.8		62.7	
		UN organizations	80.0		80.0		80.0	
		Mixed	75.0		66.7		75.0	
		Non-funded	71.4		85.7		85.7	
		Not Mentioned	29.6		45.7		42.0	
	Journal type	National	0.0	0.423	50.0	0.875	50.0	0.995
		Regional	30.8		61.5		53.8	
		International	50.3		54.4		53.7	
	Journal submission guidelines	Code of Ethics	57.4	0.001	NA	NA	60.9%	0.011
		None	27.1		NA		37.5%	

Similar results were obtained for associations between study descriptors and the likelihood of reporting access to the community/research site and seeking consent and/or assent from study participants. Articles from the biomedical field, those employing quantitative research methodologies, and those published in journals requiring a statement on ‘code of ethics’ were significantly more likely to report access to the community and seeking informed consent compared to their counterparts. Additionally, studies which reported national or regional sources of funds and those that did not mention funding sources were significantly less likely to report obtaining informed consent from study participants than their counterparts.

## Discussion

Ethical research conduct is a corner stone of research practice particularly when the research participants include populations affected by emergencies or disasters such as wars and political violence. This review of reports of ethical research conduct in studies conducted with refugees and war-affected populations is the first of its kind in the relevant literature and provides a benchmark for future research. Our findings indicate close to seven-fold increase in the number of publications on war-affected populations in the Arab region since the beginning of the 21st century, with the largest increase being in the past few years following the civil uprisings that started in 2011. Close to half of the studies included in our review involved collaborations with international researchers, with 20 and 10% of the first authors’ affiliations being from the US and the UK respectively. Armed conflicts and humanitarian assistance are increasingly becoming of international relevance, and the need for evidence and relevant data to guide humanitarian assistance is mounting.

Overall, half of the studies reviewed reported institutional approval, and a comparable percentage reported obtaining informed consent and/ or assent from participants. Owing to the lack of similar research on refugees and war-affected populations, it is difficult to compare our findings to the literature. Higher proportions of IRB approval and patient consent were reported in review studies from the medical field, with 71 and 66%, respectively, being conveyed in clinical papers appearing in anesthesia journals in 2003 [18] and 69 and 58%, respectively, being conveyed in general medical journals in 2006 [19]. Barriers to securing institutional approval are many and vary by time, place, and the context of the research itself. Al-Ahmad and colleagues [14] reviewed national research ethics regulation in Middle Eastern Arab countries and note an overall lack of adequate infrastructure and capacity to provide scientific and technical guidance on research ethics in the region, and where available, are deficient in varying levels. Additionally, the long tedious processes of review may hinder researchers and investigators who are eager to collect data in a timely manner, particularly in such contexts of wars and uncertainties where prompt humanitarian or emergency response is much needed. This may explain the drop in the reporting of ethical research conduct in 2010–11 in our study, when political upheavals started in the region and were at their peak in several Arab counties.

In our study, overall 46% of the papers did not report informed consent/ and or assent from the research participants. Although informed consent is at the center of ethical research conduct, its emphasis on individual autonomy may arguably be an imperfect means of protection from research related harms. Limiting informed consent to the individual overlooks the fact that persons make their decisions in relation to or considering others who are significant to them, such as family members or people in their social networks. This is the case in collectivist societies of the Arab world. Also as mentioned earlier, the sociopolitical and the suboptimal humanitarian conditions which people live in, such as contexts of armed conflicts, influence the decisions they make about themselves and about others taking part in research, with some implicit expectations for a compensation for their participation in the study.

Reports of ethical conduct in research in our review have increased over time. This may be attributed to the spread of awareness and the gradual attempts to institutionalize research ethics regulation and the increasing requirements of funding agencies and journal editors for ethical oversight [23]. Adherence was more noted when research was biomedical in nature compared to social research or public health, when it followed the quantitative methodology compared to the qualitative, when it involved international collaboration, and when published in journals that require statements on ethical declarations. It was least noted when the research was funded by national sources or published in national journals. The latter finding reinforces earlier observations of gaps in the knowledge base of national researchers and the suboptimal culture of ethics regulation in the Global South, including countries of the Arab region [14, 15, 24]. Our finding that greater attention to ethical guidelines is reported in biomedical research compared to other disciplines is not surprising. Clinical studies are often likely to be perceived as potentially ‘more harmful’ than social science research, despite the well documented ethically controversial earlier behavioral studies in the USA. The debate about whether the traditional orientation of the current ethics regulation adopted by ethics review boards can adequately meet the needs for social science research continues until today [25]. Given the different epistemological (how knowledge is produced) and ontological (worldview) assumptions between the two, standards adopted for the biomedical review may be inappropriate for the social sciences [26].

Our study findings need to be considered in light of certain limitations. Although our review covered a wide selection of search engines that aimed to thoroughly capture research output on refugees and war-affected populations in the Arab region, the search did not include book chapters or output from the grey literature, such as reports and publications by NGOs and humanitarian agencies. Yet, one may argue that inclusion of the ethical criteria used to guide the research is less likely to be a requirement for non-refereed publications and, hence, our findings are likely to be conservative estimates of the extent of deficiencies in the reporting of ethical research practices. On the other hand, a waiver of informed consent may have been granted in some of the research involving no more than minimal risk, or in sensitive research where consent documents may identify its participants. In such cases, the waiver of informed consent could have been unreported by the authors and hence counted as missing. Furthermore, it is not clear from this study whether IRB approval and informed consent were obtained but not reported – particularly that not all the journals require statements on ethical declaration, or whether they were obtained and reported but not adequately exercised in the field. The assessment of potential risks and burdens to participants, including unintended exploitation, unrealistic expectations and stigmatization [27], although being key elements of ethical research conduct and crucial to our understanding of the extent to which ethics in research is adhered to, is not achievable in scoping reviews. Unintended exploitation is particularly a concern in humanitarian crises, where study subjects are likely to interpret participation as being linked to provision of assistance and hence may unwillingly consent to participate [28].

## Conclusion

In conclusion, ethical research conduct appears to be underreported in publications involving refugees and war-affected populations when compared to those conducted with participants in biomedical research, with evident variations by study composition of research participants, research discipline, methodology, author collaboration, funding sources and journals requirements on ethical declaration. The heightened vulnerability of populations caught in conflict, the increased engagement of humanitarian agencies in data collection and the lack of local capacities to monitor research ethics are likely to compromise the benefit-harm ratio for the research participants and for conducting research in crisis settings [28]. Research ethics in humanitarian settings need to be seen as much more than a mechanism to obtain ethical approval for research [7]. This paper is a call for funding agencies, international organizations and relief agencies, national researchers and collaborators, and journal editors to be vigilant and play a stronger role in promoting and enhancing the practice of ethical research conduct in conflict settings and be transparent in reporting it.

## Additional file

Additional file 1: Inclusion and exclusion criteria. (DOCX 14 kb)

## Abbreviations

IDPs: Internally displaced populations
IRBs: Institutional Review Boards
RECs: Research Ethics Committee
