# Supplementary material for: A scoping review of reporting ‘Ethical Research Practices’ in research conducted among refugees and war-affected populations in the Arab world
Source: BMC Med Ethics. 2018 May 15;19:36. doi: 10.1186/s12910-018-0277-2 (PMC5952584; doi:10.1186/s12910-018-0277-2)
Supplement: Supplementary file 1 — Inclusion and exclusion criteria. (DOCX 14 kb) [file 12910_2018_277_MOESM1_ESM.docx]

**Additional file 1**

**Inclusion and exclusion criteria**

- Inclusion criteria:

- Articles published between January 2000 and December 2013
- On populations hosted in one of the 22 Arab countries
- On refugee or internally displaced populations
- Human- and population- based studies e.g. surveys, interviews, open ended questions, or focus groups.
- In English

- Exclusion criteria:

- Clinical studies

**Search Keywords**

(Refugee OR “Internally displaced” OR “Externally displaced” OR “forced migr* OR “war”)

AND

(Algeria OR Bahrain OR Comoros OR Djibouti OR Egypt OR Iraq OR Jordan OR Kuwait OR Lebanon OR Libya OR Mauritania OR Morocco OR Maroc* OR Oman OR Palestine OR “West Bank” OR Gaza OR “Occupied Pal* OR Qatar OR “Saudi Arabia” OR KSA OR Somalia OR Sudan OR Syria OR Tunisia OR Tunis OR “United Arab Emirates” OR UAE OR Yemen OR “Middle East and North Africa” OR “Eastern Mediterranean Region” OR “Arab countries” OR “Arab world” OR “Arab region” OR “North Africa”)
